# Genomic analysis of penicillin-binding proteins and recombination events in an emerging amoxicillin- and meropenem-resistant PMEN3 (Spain^9V^-3, ST156) variant in Taiwan and comparison with global descendants of this lineage

**DOI:** 10.1128/spectrum.01840-23

**Published:** 2023-11-06

**Authors:** Yi-Yin Chen, Hsin Chi, Wei-Chao Liao, Shiao-Wen Li, Yu-Ching Yang, Ho-Chen Lin, Hsiao-Pei Chang, Yi-Jiun Pan, Ruei-Lin Chiang, Yu-Chia Hsieh

**Affiliations:** 1 Department of Pediatrics, Chang Gung Children’s Hospital, Chang Gung Memorial Hospital, Chang Gung University, College of Medicine, Taoyuan, Taiwan; 2 Department of Medicine, MacKay Medicine College, New Taipei, Taiwan; 3 Department of Pediatrics, MacKay Children’s Hospital and MacKay Memorial Hospital, Taipei, Taiwan; 4 Department of Medical Research, MacKay Memorial Hospital, Taipei, Taiwan; 5 Molecular Medicine Research Center, Chang Gung University, Taoyuan, Taiwan; 6 Department of Life Sciences, National University of Kaohsiung, Kaohsiung, Taiwan; 7 Department of Microbiology and Immunology, School of Medicine, College of Medicine, China Medical University, Taichung, Taiwan; Taichung Veterans General Hospital, Taichung, Taiwan

**Keywords:** clonal shift, antibiotic resistant, non-vaccine serotype, β-lactam resistant, PMEN3 variant, Streptococcus pneumoniae

## Abstract

From 2008 to 2020, the Taiwan National Notifiable Disease Surveillance System database demonstrated that the incidence of non-vaccine serotype 23A invasive pneumococcal disease (IPD) approximately doubled. In this study, 276 non-repetitive pneumococcal clinical isolates were collected from two medical centers in Taiwan between 2019 and 2021. Of these 267 pneumococci, 60 were serotype 23A. Among them, 50 (83%) of serotype 23A isolates belonged to the sequence type (ST) 166 variant of the Spain^9V^-3 clone. Pneumococcal 23A-ST166 isolates were collected to assess their evolutionary relationships using whole-genome sequencing. All 23A-ST166 isolates were resistant to amoxicillin and meropenem, and 96% harbored a novel combination of penicillin-binding proteins (PBPs) (1a:2b:2x):15:11:299, the newly identified PBP2x-299 in Taiwan. Transformation of the *pbp1a*, *pbp2b*, and *pbp2x* alleles into the β-lactam-susceptible R6 strain revealed that PBP2x-299 and PBP2b-11 increased the MIC of ceftriaxone and meropenem by 16-fold, respectively. Prediction analysis of recombination sites in PMEN3 descendants (23A-ST166 in Taiwan, 35B-ST156 in the United States, and 11A-ST838/ST6521 in Europe) showed that adaptive evolution involved repeated, selectively favored convergent recombination in the capsular polysaccharide synthesis region, PBPs, *murM*, and *folP* genome sites. In the late 13-valent pneumococcal conjugate vaccine era, PMEN3 continuously displayed an evolutionary capacity for global dissemination and persistence, increasing IPD incidence, leading to an offset in the decrease of pneumococcal conjugate vaccine serotype-related diseases, and contributing to high antibiotic resistance.

A clonal shift with a highly β-lactam-resistant non-vaccine serotype 23A, from ST338 to ST166, increased in Taiwan. ST166 is a single-locus variant of the Spain^9V^-3 clone, which is also called the PMEN3 lineage. All 23A-ST166 isolates, in this study, were resistant to amoxicillin and meropenem, and 96% harbored a novel combination of penicillin-binding proteins (PBPs) (1a:2b:2x):15:11:299. PBP2x-299 and PBP2b-11 contributed to the increasing MIC of ceftriaxone and meropenem, respectively. Prediction analysis of recombination sites in PMEN3 descendants showed that adaptive evolution involved repeated, selectively favored convergent recombination in the capsular polysaccharide synthesis region, PBPs, *murM*, and *folP* genome sites. In the late 13-valent pneumococcal conjugate vaccine era, PMEN3 continuously displays the evolutionary capacity for dissemination, leading to an offset in the decrease of pneumococcal conjugate vaccine serotype-related diseases and contributing to high antibiotic resistance.

## INTRODUCTION

Pneumococcal disease remains an important health threat, given the increase in the incidence caused by serotypes not included in the pneumococcal conjugate vaccine (PCV).

After the national 13-valent pneumococcal conjugate vaccine (PCV13) immunization program in 2013 (1), serogroup 15 was the most common type associated with invasive pneumococcal disease (IPD) from 2015 to 2017, accounting for 11%–12% (61/524 to 56/456) of the total annual IPD (2), and most serotype 23A (8%–9% of total IPD) isolates belonged to sequence type (ST) 338 (3, 4) in Taiwan. In 2018, serotype 23A (11.3% of total IPD) abruptly increased as the most common type of pneumococcal disease (5), together with a switch from a previously meropenem-susceptible ST338 to meropenem-resistant ST166 in two multicenter studies (3, 6). ST166 is a single-locus variant of the Spain^9V^-3 clone (ST156), which is also called the PMEN3 lineage, showing reduced susceptibility to penicillin. It originated from Spain in the 1980s and attained global spreading thereafter before the use of PCV (7, 8). The PMEN3 lineage carries the *rlrA* pilus islet to contribute to adherence, stimulate the host immune response, facilitate nasopharyngeal colonization, and increase virulence, as reflected by the significant association between the PMEN3 lineage and invasive diseases (9, 10). Before the use of PCV, the pilated PMEN3 lineage was associated with several serotypes, including 6B, 14, 19A, 19F, 23F, and 24F (11). Some strains harbored tetracycline (*tet*) and macrolide (*ermB*, *mef*, and *mrsD*) resistance genes, and some strains also had *folP* insertion (conferring sulfamethoxazole resistance), mutation of *folA* (conferring trimethoprim resistance), and mutation of *parC* and *gryA* (conferring fluoroquinolone resistance) during the evolutionary process to increase fitness for antibiotic pressure (12 – 14). After the widespread use of PCV13, the PMEN3 lineage 9V-ST156-PBP:15:12:18 in the United States underwent a capsular switch event with 35B-ST558-PBP:4:7:7, cotransferring bilateral flanking penicillin-binding protein (PBP) genes *pbp1a* and *pbp2x* to become 35B-ST156 with PBP type 4:12:7 for persistent penicillin susceptibility (MIC ≤ 2 mg/L) emergent strain (15). In Spain, France, and Portugal, PMEN3 evolved into two main serotype 11A vaccine escape variants (11A-ST838 and 11A-ST6521) after four to seven fragment recombination events (16). The 11A-ST838, a single-locus variant of ST156, and 11A-ST6521, a double-locus variant of ST156, harbored a new PBP type (15:76:new1), showing resistance to penicillin and amoxicillin (MIC ≥ 2 mg/L). In Taiwan, we hypothesized that the pilated PMEN3 lineage Spain^9V^-ST156 transformed into 23A-ST166, together with changes in PBPs, to continue the spread of the PMEN3 lineage under selective pressure.

In this study, we reported the dynamics of national serotype 23A IPD between 2008 and 2020 and launched a whole-genome initiative to characterize the 23A-ST166 clone prevalent in Taiwan and its relationship with the ancestral 9V-ST156 clone and other PMEN3 descendants, including the 35B-ST156 variant in the United States and 11A-ST838/ST6521 variant in Europe, to investigate global diversification of PMEN3 under PCV. The effect of the new combination of PBP profile of 23A-ST166 on anti-β-lactam susceptibility was assessed by a series of transformation studies.

## MATERIALS AND METHODS

### Surveillance data, bacterial collection, culture, and antibiotic susceptibility analysis

The total incidence of IPD between 2008 and 2020 was obtained from the Taiwan Centers for Disease Control (TCDC) IPD national surveillance system (1). Furthermore, from January 2019 to May 2021, a total of 242 and 34 non-repetitive pneumococcal clinical isolates were collected from 3700-bed CGMH-Linkou and 1130-bed MMH-Taipei, respectively, in Taiwan. Most isolates were collected from the sputum (*n* = 92, 33.3%), followed by the ears, nose, and throat (ENT, *n* = 61, 22.1%); pus (*n* = 58, 21%); and blood (*n* = 21, 7.61%). Pneumococci were cultured at 37°C in a 5% CO_2_ incubator in Todd-Hewitt broth supplemented with 0.5% yeast extract or on blood agar plates (5% defibrinated sheep blood). Standard Latex agglutination, Quellung reactions, and multilocus sequence typing (MLST) (17) were used to confirm the presence of 23A-ST166 pneumococcus. The antibiotic susceptibilities of pneumococci to penicillin, amoxicillin, ceftriaxone, meropenem, ciprofloxacin, and levofloxacin were determined by the microdilution method described by the Clinical and Laboratory Standards Institute (CLSI) (18). All drugs were purchased from Sigma-Aldrich (Sigma-Aldrich, USA).

### Whole-genome sequencing and assembly

To study the evolutionary relationships of 23A-ST166 in Taiwan, we used 52 *S*. *pneumoniae* clinical isolates to perform whole-genome sequencing (WGS), including 50 23A-ST166 isolates collected during 2019–2021, and one each of 9V-ST156 and 9V-ST166 identified in our previous study (19, 20). Genomic DNA of pneumococci was extracted using the QIAamp DNA Mini Kit (QIAGEN, Germany), and sequencing libraries were prepared for WGS using NEBNext Ultra II DNA Library Prep Kits (Illumina, USA) with Illumina MiSeq for 600 cycles (2 × 150 bp paired-end). The raw read quality assessment was performed using FastQC version 0.11.3. The contig was assembled using SPAdes version 3.13.0 (21) and evaluated using QUAST (22) with an N_50_ length of 87,487 bp. All WGS data in this study were deposited in the Sequence Read Archive (SRA) database under the BioProject PRJNA852704 (Table S1).

### Comparing genomic variation of PMEN3 descendants from different countries

The genomic characteristics of 23A-ST166 isolates from Taiwan were compared with those of isolates from European countries and the United States. WGS data were downloaded from the NCBI SRA database (BioProject: PRJEB24965, PRJNA284954, and PRJEB32798). Draft genome data, from 14 35B-ST156 and 49 11A-ST838/ST6521 isolates, were collected for subsequent analysis (15, 16).

### Predicting recombination sites by phylogenetic analysis

WGS trimmed reads were aligned against the ATCC-700671 (9V-ST156, GenBank accession no: CP099641) as described previously (2), and the resulting phylogenetic tree, isolate metadata, core genome SNPs, and recombination sites were visualized by using Phandango version 1.3.0 (23).

### Penicillin-binding protein profiles

To compare the sequences of the transpeptidase regions of the *pbp1a*, *pbp2b*, and *pbp2x* genes of 23A-ST166, *in silico* PBP profiles were determined using BLASTX by aligning the assembly nucleotide contig with the U.S. CDC PBP database (https://www.cdc.gov/streplab/pneumococcus/mic.html).

### Penicillin-binding protein transformation

The non-encapsulated laboratory strain R6, an amoxicillin-susceptible pneumococcus (MIC 0.016 mg/L), was used as the recipient in PBP transformation studies. The entire *pbp1a*, *pbp2b*, and *pbp2x* genes of the 23A-ST166 clinical isolates were PCR amplified and then cloned into pJET1.2/blunt (Thermo Fisher, USA) according to the manufacturer’s instructions (2). The *pbp*-containing plasmid was transformed into the *S. pneumoniae* R6 strain using CSP-1 (2) and then spread on Mueller-Hinton agar containing 5% sheep blood and different concentrations of amoxicillin. After 24 h of incubation, colonies were selected from plates containing the highest amoxicillin concentration. The *pbp* gene of the transformants was PCR amplified and Sanger sequenced. The MICs of antibiotics in the selected transformants were analyzed according to CLSI guidelines.

### Antimicrobial-resistant gene detection

To detect the presence of antimicrobial resistance genes, the Comprehensive Antibiotic Resistance (CARD, https://card.mcmaster.ca/) database was used to search for assembly contigs of the 23A-ST166 isolates. The mutations of *folA*, *gyrA*, and *gyrB* and the insertion of *folP* for all 23A-ST166 were determined using BLASTX with the reference sequence from GenBank (*folA*, AE007317, 1412861–1413367; *gyrA*, AAK99902.1; *gyrB*, AAK99519.1; and *folP*, AE007317, 268022–268966).

### Statistical analysis

SPSS 15.0 for Windows (Statistical Package for Social Sciences, Chicago, IL, USA) was used for statistical analysis. Statistical comparisons of the incidence were performed using Poisson distribution with 95% confidence intervals. Differences were statistically significant at *P* < 0.05.

## RESULTS

### Serotype replacement with the increase of serotype 23A-associated IPD from 2010 to 2020

**Fig 1:**
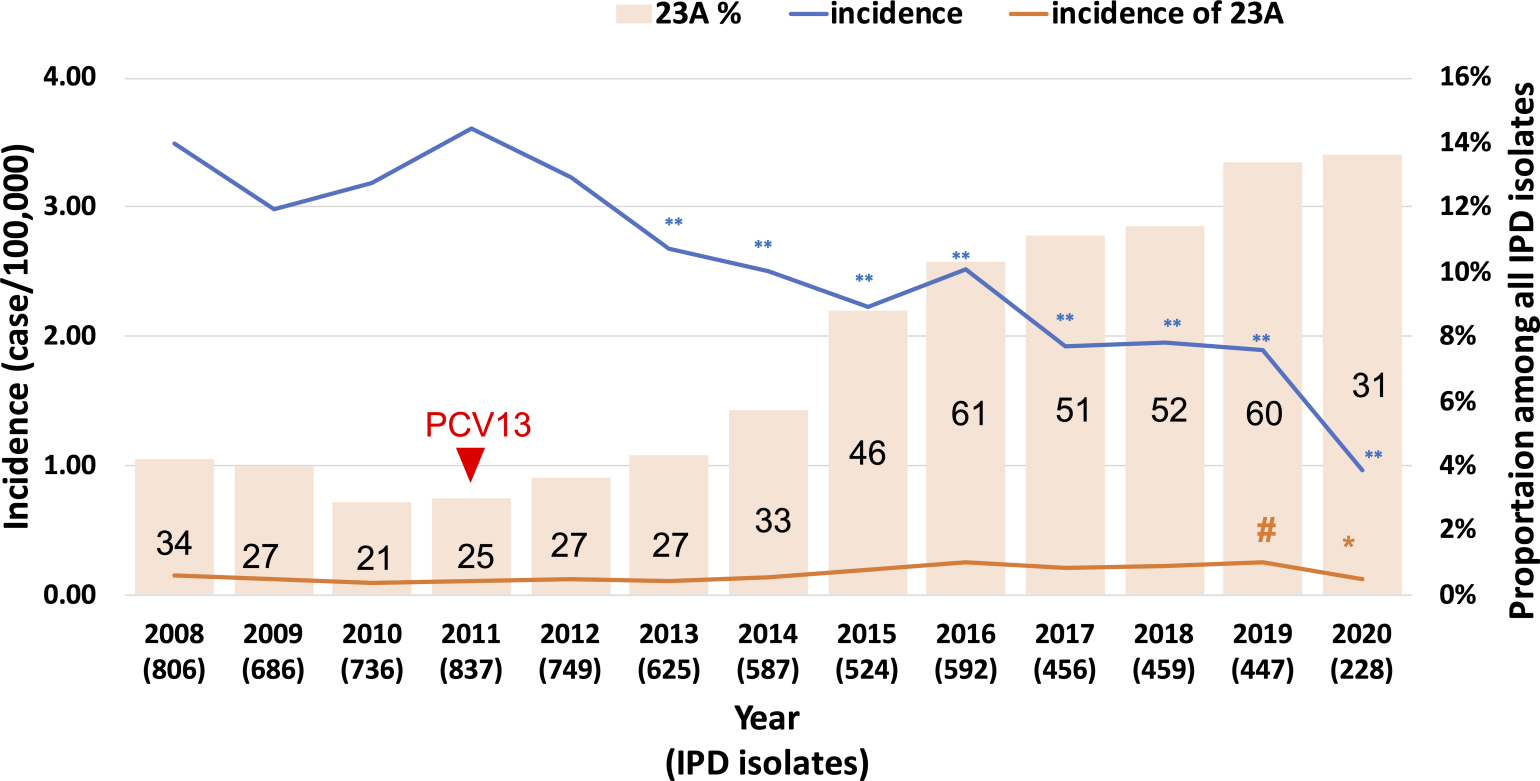
Incidence (lines) of IPD and proportions (bars) of IPD cases caused by serotype 23A during 2008–2020 post PCV13 immunization program in Taiwan (red triangle in 2011). Surveillance data from Taiwan Centers for Disease Control IPD Surveillance System show that the incidence (orange line) and percentage (orange bar) of serotype 23A increased during 2008–2020. Post PCV13 vaccination, the non-vaccine serotype with the highest incidence was serotype 23A between 2018 and 2020 (^#^ *P* = 0.011 in 2019 and *P = 0.05 in 2020, compared with 2008). In contrast, the total incidence of IPD (blue line) significantly decreased between 2013 and 2019 (**P < 0.001, compared with 2008). The black numbers indicate the numbers of IPD-causing serotype 23A. IPD, invasive pneumococcal disease; PCV13, 13-valent pneumococcal conjugate vaccine.

Surveillance data from the TCDC IPD surveillance system showed that the total incidence of IPD significantly decreased from 3.5 episodes/100,000 persons/year in 2008 to 0.97 episodes/100,000 persons/year in 2020 after the PCV13 vaccination program (*P* < 0.001). However, the incidence of IPD caused by serotype 23A increased from 0.15 episodes/100,000 persons/year in 2008 to 0.25 episodes/100,000 persons/year in 2019 (*P* = 0.011) and then decreased to 0.13 episodes/100,000 persons/year in 2020 (Table S1; Fig. 1). The prevalence of serotype 23A among all IPD increased from 4.2% (*n* = 34) in 2008 to 13.4% (*n* = 60) and 13.8% (*n* = 31) in 2019 and 2020, respectively. Serotype 23A was the top non-vaccine serotype causing IPD between 2008 and 2011 but was replaced by serotype 15A between 2012 and 2017. Subsequently, serotype 23A returned to the most common non-vaccine serotype in 2018 (Table S1) and became the most dominant IPD-causing pneumococcal serotype by 2020 (more prevalent than other vaccine serotypes). The incidence of serotype 23A causing IPD increased annually between 2008 and 2019 and then decreased in 2020 owing to the COVID-19 pandemic. In February 2020, the implementation of a mask-wearing policy in Taiwan resulted in a decrease in the incidence of IPD-causing serotype 23A in 2020. However, serotype 23A remained the predominant serotype causing IPD in 2020. Before 2014, the genotype of serotype 23A was ST338; however, this clone shifted to ST166 by 2018 (6). Therefore, we collected pneumococci from two tertiary medical centers in 2019 and 2021 to study the major circulating ST166 serotype 23A pneumococci, a PMEN3 clone variant, in Taiwan after 2019.

### Clonal expansion of amoxicillin- and meropenem-resistant 23A-ST166 strains

A total of 276 non-repetitive pneumococcal clinical isolates were collected from the CGMH and MMH between 2019 and 2021. Among the isolates, serotype 23A (*n* = 60, 21.7%) was the most common, followed by 3 (11.2%), 15A (9.1%), and 19A (6.2%). The 23A pneumococcal isolates were collected from the ears, nose, and throat (ENT; *n* = 24, 40%); sputum (*n* = 18, 30%); and pus (*n* = 18, 30%). The ST of 60 serotype 23A pneumococcal isolates was determined by using the MLST method. Among the 60 isolates, most were identified as 23A-ST166 (*n* = 50, 83.3%), consisting of 52% (*n* = 26) in 2019, 40% (*n* = 20) in 2020, and 8% (*n* = 4) in 2021. The MIC_90_ (MIC for 90% of the strains) values for penicillin, amoxicillin, ceftriaxone, and meropenem were 2, 8, 2, and 1 mg/L, respectively. The MIC_50_ and MIC ranges are listed in Table 1. In the meningitis criteria, the non-susceptibility criteria to penicillin and ceftriaxone were 100% and 98%, respectively, whereas in the non-meningitis criteria, these were 0% and 30%, respectively. Furthermore, the non-susceptibility rates to amoxicillin and meropenem were 100%, whereas those to ciprofloxacin and levofloxacin were less than 10% (Table 1; Table S2). In addition, the ST of the other 10 serotype 23A isolates were ST338 (*n* = 4), ST15119 (*n* = 3), ST15236 (*n* = 1), ST15237 (*n* = 1), and ST63 (*n* = 1). The MICs for penicillin and ceftriaxone are listed in Table S3.

**TABLE 1:** Antimicrobial activity of six antimicrobial agents against 50 *Streptococcus pneumoniae* 23A-ST166 between 2019 and 2021

Antimicrobial agent	MIC_50_ (mg/L)	MIC_90_ (mg/L)	Range (mg/L)	Range % of non-susceptible isolates	
				Meningitis	Non-meningitis
Penicillin	2	2	1–2	100^*a*^	0^*b*^
Amoxicillin	4	8	4–8	−^*e*^	100
Ceftriaxone	1	2	0.5–8	98^*c*^	30^*d*^
Meropenem	1	1	0.5–2	−^*e*^	100
Ciprofloxacin	1	1	0.5–2	−^*e*^	8
Levofloxacin	1	1	0.5–4	−^*e*^	2

^*a*^ For penicillin, we defined the criteria for meningitis as MIC < 0.06 mg/L, susceptible, and MIC > 0.12 mg/L, non-susceptible.

^*b*^ For penicillin, we defined the criteria for non-meningitis as MIC < 2 mg/L, susceptible, and MIC > 4 mg/L, non-susceptible.

^*c*^ For ceftriaxone, we defined the criteria for meningitis as MIC < 0.5 mg/L, susceptible, and MIC > 1 mg/L, non-susceptible.

^*d*^ For ceftriaxone, we defined the criteria for non-meningitis as MIC < 1 mg/L, susceptible, and MIC > 2 mg/L, non-susceptible.

^*e*^ Undefined.

**TABLE 2:** Penicillin-binding protein transformation study^*a*^

Recipient	Transforming DNA	Transformant name	Source	Colony formation	MIC (mg/L) of transformants						Integration of altered PBP in transformants	PBP profile 1a:2b:2x
					Penicillin	Meropenem	Amoxicillin	Ceftriaxone	Ciprofloxacin	Levofloxacin		
9V-ST156	NA	9V-ST156-WT	TW	ND	1	0.5	0.5	1	1	1	ND	15:12:18
9V-ST166	NA	9V-ST166-WT	TW	ND	1	0.5	1	1	1	0.5	ND	15:12:18
23A-ST166	NA	23A-WT	TW	ND	2	1	8	2	1	1	ND	15:11:299
R6	NA	R6-WT	ATCC	ND	0.016	0.016	0.016	0.03	1	1	ND	196:0:2
R6	*pbp1a*	R6–1A	−	−	ND	ND	ND	ND	ND	ND	ND	ND
R6	*pbp2b*	R6–2B	−	−	ND	ND	ND	ND	ND	ND	ND	ND
R6	*pbp2x*	R6–2X	TW	+	0.06	0.03	0.03	0.5	1	0.5	Yes, PBP2x	196:0:299
R6–2X transformants	*pbp2b*	R6–2X-2B	TW	+	0.25	0.5	0.25	0.5	1	0.5	Yes, PBP2b	196:11:299
R6–2X transformants	*pbp1a*	R6–2X-1A	−	−	ND	ND	ND	ND	ND	ND	ND	ND
R6–2X-2B transformants	*pap1a*	R6–2X-2B-1A	TW	+	2	1	4	2	1	0.5	Yes, PBP1a	15:11:299

^*a*^ NA, not applicable; TW, Taiwan; ATCC, American Type Culture Collection; ND, not done; PBP, penicillin-binding protein; +, positive for colony formation on amoxicillin agar plates within 24 h of incubation; −, negative for colony formation on amoxicillin agar plates within 24 h of incubation.

### PBP combination profile with novel PBP2x-299 in 23A-ST166

Among the 23A-ST166 isolates collected from 2019 to 2021, 96% (*n* = 48) strains had a new combination of PBP allelic profiles 15:11:299 for PBP1a, 2b, and 2x. PBP2x-299 was newly identified in Taiwan. 23A-ST166 harbored PBP1a-15, which was identical to the original PMEN3 (PBP1a:2b:2x = 15:12:18). PBP2b-11 of 23A-ST166 was the same as the multidrug-resistant 19A-ST320 (PBP1a:2b:2x = 13:11:16), and had 10 amino acid (aa) substitutions in the 590–641 region (A591S, G596P, N605D, L608T, A618G, D624G, Q627E, T629N, S639T, and D640E) compared with PBP2b-12 of PMEN3. The new PBP2x-299 of 23A-ST166 had eight aa changes (I366M, A369V, and E378D in the transpeptidase domain and an alteration of the other five residues, A507T, E705E, T709A, A711S, and E719V) compared with PBP2x-18 of PMEN3 (Tables S4 to S6).

### PBP variation of 23A-ST166 increases the β-lactam non-susceptibility for *S. pneumoniae* R6

Considering that the MIC of amoxicillin in the 23A-ST166 isolates increased to 8 mg/L (Table 2; Table S2) compared with the MIC 0.5 mg/L of amoxicillin for 9V-ST156 and 9V-ST166 collected from 2001 and 2002 in Taiwan, we analyzed the effect of PBP variants on antimicrobial drug susceptibility of β-lactam, using amoxicillin as a selection antibiotic. The genomic DNA of 23A-ST166, with the highest MIC = 8 mg/L for amoxicillin in our study, was chosen as the PCR template. The MIC of R6−2X transformants for penicillin, amoxicillin, and meropenem increased by two- to four-fold, while largely increasing by 16-fold, from 0.03 mg/L to 0.5 mg/L for ceftriaxone. The MIC of R6–2X-2B transformants for amoxicillin, meropenem, and penicillin increased by 8-, 16-, and 4-fold, respectively. The MIC of R6–2X-2B transformants for ceftriaxone did not change. The MICs of the four β-lactam antibacterial drugs for the R6–2X-2B-1A transformant increased 2–16-fold, which was similar or the same level as that of 23A-ST166 (Table 2). Considering that there was no colony formation, we could not evaluate the MICs for transformants of R6−1a, R6−2b, and R6−2x-1a (Table 2). DNA sequence analysis of transformants in each transformation step confirmed that all amino acid substitutions occurred in the PBP1a, PBP2b, and PBP2x transpeptidase domains in the recipient strain.

### Other antimicrobial resistance genes and pilus determinants

Among the whole-genome sequenced 23A-ST166 isolates, all had *ermB*, conferring resistance to macrolide antibiotics, *tetM*, conferring resistance to tetracycline, and a *folA* mutation (I100L substitution) known to decrease bacterial trimethoprim susceptibility. Only 4 of the 50 isolates had a *folP* insertion (two codon insertions between bases 168 and 201), conferring resistance to sulfamethoxazole; one isolate had a *gyrA* mutation, conferring resistance to fluoroquinolone, and none carried mutations in *parC* and *gyrB*. In addition, all but one 23A-ST166 isolate had pilus determinants (*rlrA* islets) in its genome (Table S2).

### Phylogenetic tree analysis of 23A-ST166 compared with its ancestor clone 9V-ST156

**Fig 2:**
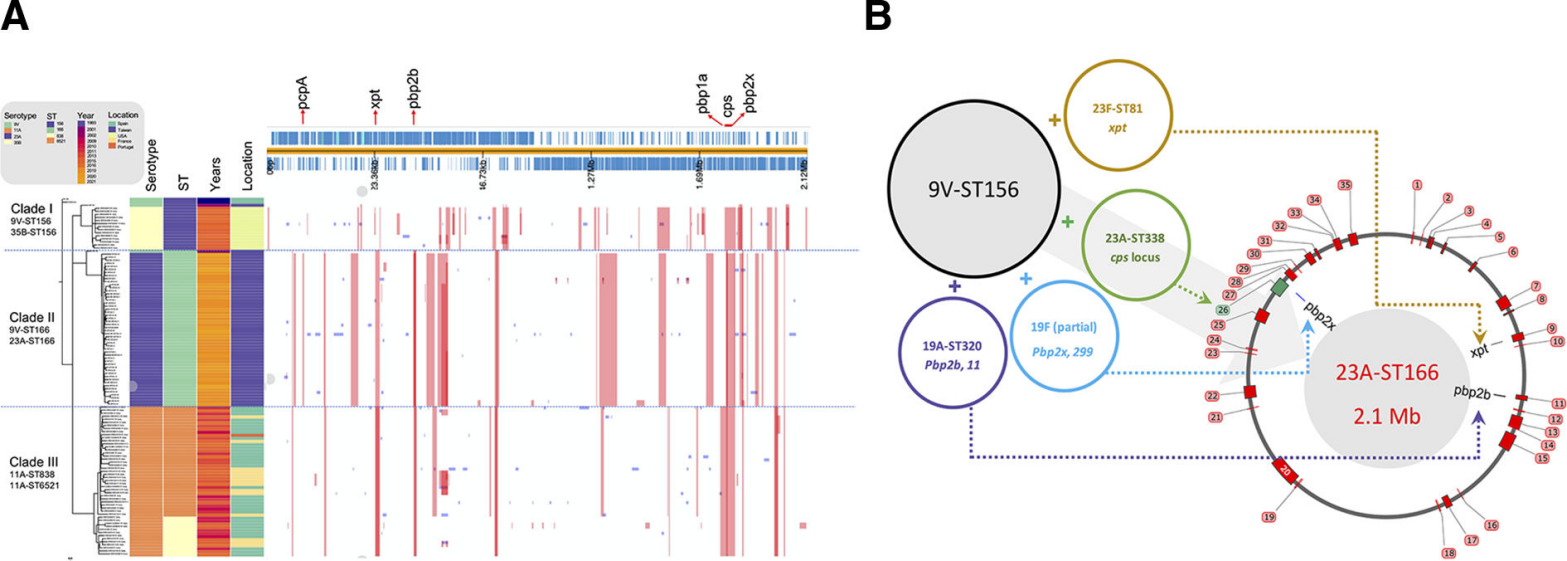
(**A**) Phylogenetic tree and recombination sites of 50 *Streptococcus pneumoniae* 23A-ST166 isolates, one each of 9V-ST156 and 9V-ST166 isolates in Taiwan, and 14 35B-ST156 isolates in the United States and 49 11A-ST838/ST6521 isolates in Europe (Spain [*n* = 29], France [*n* = 19], and Portugal [*n* = 1]). The phylogenetic tree compares these 115 isolates against the reference strain, *S. pneumoniae* Spain^9V^-ST156 ATCC-700671 (GenBank accession no. CP099641). Recombination positions are based on the 2,116,814 bp of the reference strain. The scale bar indicates nucleotide substitutions per site. (**B**) Putative evolutionary of *S. pneumoniae* from 9V-ST156 to 23A-ST166. Black, receptor 9V-ST156; brown, putative *xpt* donor; green, putative *cps* locus donor; light-blue, putative partial *pbp2x* donor; purple, putative *pbp2b* donor. The coordinates refer to the ones of genome of strain ATCC-700671 (GenBank accession no. CP099641). The approximate recombination size is indicated in red color.

Using ATCC-700671 (9V-ST156, GenBank accession no: CP099641) as an outgroup, we constructed a whole-genome phylogenetic tree for the 23A-ST166, 9V-ST156, and 9V-ST166 isolates (collected from Taiwan) (Fig. 2A). Compared with the original 9V-ST156 from Spain, 9V-ST156 circulated in Taiwan recombined at 10 regions and at an additional eight regions to be 9V-ST166, before the use of PCV13. Compared with 9V-ST166, there were 27 specific recombination events in all 23A-ST166 isolates, including several genes that might contribute to pneumococci evolution: (i) the capsule polysaccharide (*cps*) locus, which was closely related to that of an 23A-ST338 isolate (99.81% identity), at position 1797968–1834794; (ii) *xpt*, with 100% identity to that of 23F-ST81, at position 427406–444217; (iii) *pbp2b*, with 100% identity to that of 19A-ST320, at position 576806–588951; (iv) *pbp2x*, with 99.4% identity to that of a 19F isolate named S-161 (GenBank accession no. CMY36221), at position 1804245–1837514; (v) cell surface choline binding protein PcpA (*pcpA*) at position 143506–148718; (vi) putative late competence protein (*comF*) at position 64073–66351; and (vii) polyamine transport protein D (*potD*) at position 865807–866612 (Table S7; Fig. 2B).

### Global dissemination of PMEN3 (Spain^9v^-ST156) variant

**Fig 3:**
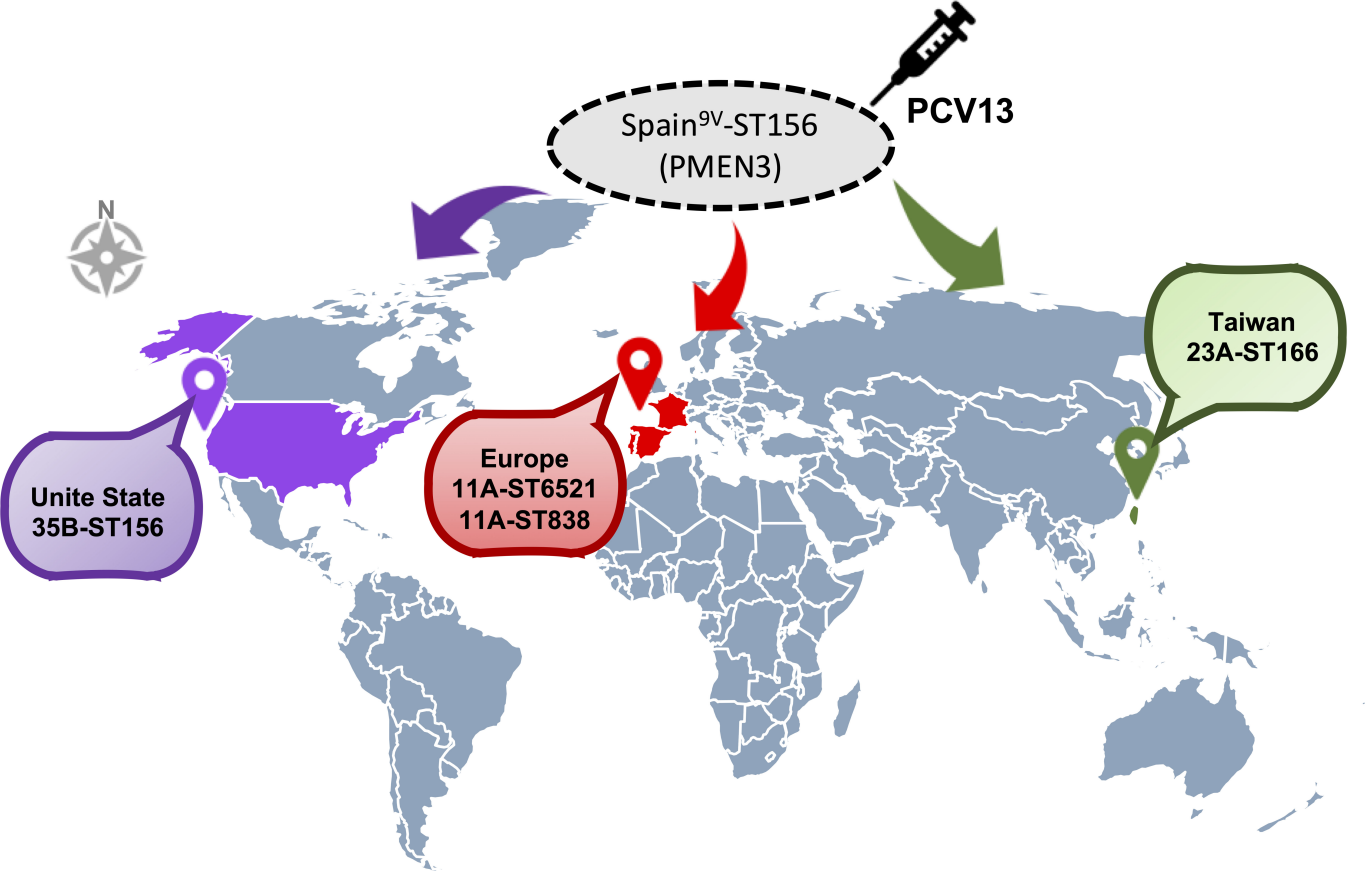
Dissemination of the Spain^9V^-ST156 (PMEN3) variant clone following PCV13 vaccination. The map was created using the amCharts Pixel Map Generator.

To study how PMEN3 lineages have evolved globally (Fig. 3), we constructed a whole-genome phylogenetic tree. From the database, 63 WGS data of *S. pneumoniae* strains were downloaded. Among these, 13 11A-ST838 and 36 11A-ST6521 were in Europe, and 14 35B-ST156 were in the United States. PMEN3 isolates were divided into the following three clades: (i) all 35B-ST156 isolates clustered together with 9V-ST156, (ii) all 23A-ST166 isolates clustered together with 9V-ST166, and (iii) all serotype 11A clustered together (Fig. 2A). Compared with the original 9V-ST156 from Spain, 35B-ST156 and 11A-ST838/ST6521 had 25 and 7 unique recombination events, respectively. Several recombination events related to evolution, including the bacterial serotype switch-related recombination in the CPS region (in all PMEN3 variants), antibiotic susceptibility-related recombination in genes such as *pbp1a*, *pbp2b*, *pbp2x*, *murM*, and *folP* conferring resistance to β-lactam and sulfamethoxazole, and recombination of transposase-related and phage-related genes that increase the fitness of pneumococci to adapt to environmental stress, were observed in most PMEN3 variants (Table S7; Fig. 2A).

## DISCUSSION

The increase in amoxicillin- and meropenem-resistant serotype 23A replacement in Taiwan was mainly mediated by the expansion of non-vaccine serotype 23A with a previously well-adapted, globally spreading antibiotic-resistant PMEN3 lineage through multiple recombination events. The 9V-ST156 and its single-locus variant ST166 had been circulating before PCV13 in Taiwan (3, 4). Serotype/capsular switch events in 23A-ST166 should have occurred between the 23A-ST338 strain as the serotype/capsular *cps23A* donor and the 9V-ST166 strain as the recipient.

Genomic surveillance of the dynamics of pneumococcal populations after the widespread use of PCV revealed that previously existing clones changed their capsular types to non-vaccine types to evade vaccine-induced immunity, continuing their spread in the community (24). These previously existing successful clones usually harbor specific virulence factors or antibiotic resistance genes to be disseminated in many countries. Intriguingly, they are adept at undergoing capsular switching events to express various capsular types. For instance, the Spain^23F^-1 clone (ST81) possesses an integrative conjugative element (ICE), MM1 phage, Na^+^-dependent ATPase Island, and TprA2/PhrA2 genomic regions, which are associated with increased colonization and virulence (25, 26). ST81 also expresses alternative types, such as 19F, 14, 6B, and 15B (27). From 2013 to 2017, we reported that the Spain^23F^-ST81 clone rapidly recombined in 12 regions and transformed into 15B/C-ST83, which became the most common penicillin- and meropenem-resistant clone in Taiwan (2). The expansion of successful clones mediated by capsular switching to non-vaccine types with high-level antibiotic resistance raises concerns regarding the historical predominance of these epidemic clones (15).

Similarly, the PMEN3 lineage in Taiwan acquired genetic variation through recombination with 23A-ST338 in the capsular synthesis region, 19A-ST320 on *pbp2b*, 23F-ST81 on *xpt*, and other multiple loci, such as *pcpA*, *comF*, and *potD*, which are associated with pneumococcal metabolism, virulence, natural competence, and antibiotic resistance development (28 – 30) for rapid adaptation to selective pressure. PBP2b-11, which originated from a pandemic multidrug-resistant 19A-ST320 clone, increased amoxicillin resistance (15) and had 10 unique changes in the 590–641 amino acid region, the key alteration associated with amoxicillin resistance (31, 32). PMEN3 was initially susceptible to amoxicillin and non-susceptible to meropenem, turning into amoxicillin- and meropenem-resistant strains after receiving *pbp2b* from 19A-ST320. We also demonstrated that the novel PBP2x-299 substantially increases the MIC of ceftriaxone. In a study of isolates belonging to the PMEN3 lineage from 31 countries between 1992 and 2012, a high frequency of recombination (*r*/*m* ratio:0.115 recombination/point mutation) and great diversity of the imported sequence (11.8 single nucleotide polymorphisms/kb of sequence imported) were found in the PMEN3 reconstruction, which accounted for their successful dissemination between countries (33). In the late PCV13 period, together with global descendants that emerged after 2015, including 35B-ST156 in the United States and 11A-ST838/ST6521 in Europe, the global landscape of PMEN3 lineages involved repeated, selectively favored convergent recombination at CPS, PBPs, *murM*, and *folP* genome sites. These results were consistent with those of a previous study that demonstrated that the most common recombination events occurred on PMEN3 located in the capsular region (adjacent to *pbp1a* and *pbp2x*), *murM*, and *pbp2b* over a 30-year period (12).

The extent of serotype replacement by non-vaccine serotypes varies between countries. Non-PCV13 serotypes such as 11A, 15A, 35B, and 23A were prevalent in Asian countries (34); 12F, 15B/C, 24, and 5 in Israel (35); 8, 35B/D, 12F, 16F, and 15B/C in South Africa (35); and 15B/C, 22F, 33F, and 35B/D in the United States (35). Furthermore, with selection by community antibiotic consumption, non-vaccine serotype pneumococci adaptively evolved with increased MIC of third-generation cephalosporins, fluoroquinolones, and carbapenems after the introduction of PCV13 (36). The emergence of multidrug-resistant pneumococci, a worrisome challenge for clinicians, may cause infections that fail to respond to commonly used antibiotics, resulting in life-threatening diseases. Serotype 23A has a low invasive disease potential, yet it frequently causes disease in immunocompromised hosts and individuals with chronic medical conditions (35). In recent years, an increase in serotype 23A has occurred not only in Asian countries (35) but also in Israel and Belgium (37, 38). Currently, 23 valent pneumococcal polysaccharide vaccines (PPV23), PCV13, PCV15, and PCV20 include serotype 23F but not serotype 23A. Genes for capsular polysaccharide synthesis between serotypes 23A and 23F are similar, except for the oligosaccharide polymerase Wzy, which results in different polymerization and divergent polysaccharide structures (39). Although typing antiserum with serotype 23F reacts slightly with serotype 23A, immunity generated after pneumococcal vaccine containing serotype 23F cannot cross-protect against serotype 23A infection. Optimization of vaccination strategies using a PCV containing serotype 23A could be of value for patients with a high risk of serotype 23A infection.

In this study, we delineated the molecular mechanism of clonal shift within serotype 23A serotype replacement in Taiwan, deconvoluted the development of the ST166 lineage evolved from its ancestral clone, and analyzed genetic variation in PMEN3’ descendants in other countries. These findings expand the current knowledge regarding the remarkable evolutionary capacity of the PMEN3 lineage. In addition, we used a transformation study to demonstrate that the new PBP2x-299 contributed to the increased MIC of ceftriaxone in *S. pneumoniae*, presumably due to the additional eight aa changes that reduce the binding affinity of ceftriaxone to PBP2x. The limitations of this study include the following: (i) we collected serotype 23A isolates from only two medical centers, which may not be sufficiently representative. However, these isolates clustered tightly and exhibited consistent recombination sites. Additional genomic differences among isolates from various regions may be limited and (ii) all isolates were obtained from non-invasive sites. It is not clear whether the most invasive serotype 23A isolates belonged to ST166. Colonization is the first step in the development of pneumococcal disease. We believe that a clonal shift from ST338 to ST166 also occurred among the invasive isolates following the use of antibiotics and PCV13.

To date, the PCV strategy has been successful in decreasing the virulent capsular type without the selection of dangerous clones. Nevertheless, the convergence of extensive resistance through multiple recombination events in pre-existing epidemic clones of *S. pneumoniae* represents an alarming threat. Restriction of antibiotic overuse to inhibit the spread of super-resistant clones, especially those causing disease in patients with chronic disease, remains an important matter of concern.

## Data Availability

The whole-genome sequence data generated during this study were deposited in the SRA database under the BioProject number PRJNA852704. De-identified patient data sets will be available from the corresponding author upon written request.

## SUPPLEMENTAL MATERIAL

Supplemental tables spectrum.01840-23-s0001.xlsx Tables S1 to S7.

ASM does not own the copyrights to Supplemental Material that may be linked to, or accessed through, an article. The authors have granted ASM a non-exclusive, world-wide license to publish the Supplemental Material files. Please contact the corresponding author directly for reuse.
